# Feasibility of a person-centred multidimensional interdisciplinary rehabilitation programme in community-dwelling people with dementia: a randomised controlled pilot trial

**DOI:** 10.1186/s12877-024-05372-9

**Published:** 2024-09-28

**Authors:** Låtta Hasselgren, Mia Conradsson, Josefine Lampinen, Annika Toots, Birgitta Olofsson, Ingeborg Nilsson, Maria Gustafsson, Nina Lindelöf, Henrik Holmberg, Yngve Gustafson, Håkan Littbrand

**Affiliations:** 1 Department of Community Medicine and Rehabilitation, Geriatric Medicine, Umeå University, Umeå, Sweden; 2 Department of Community Medicine and Rehabilitation, Occupational Therapy, Umeå University, Umeå, Sweden; 3 Department of Nursing, Umeå University, Umeå, Sweden; 4 Department of Surgical and Perioperative Science, Orthopaedics, Umeå University, Umeå, Sweden; 5 Department of Medical and Translational Biology, Umeå University, Umeå, Sweden; 6 Department of Community Medicine and Rehabilitation, Physiotherapy, Umeå University, Umeå, Sweden; 7 Department of Epidemiology and Global Health, Umeå University, Umeå, Sweden

**Keywords:** Community-dwelling, Dementia, Feasibility study, Interdisciplinary, Rehabilitation

## Abstract

**Background:**

A team-based, individualised rehabilitation approach may be required to meet the complex needs of people with dementia. This randomised controlled pilot trial evaluated the feasibility of a person-centred multidimensional interdisciplinary rehabilitation programme for community-dwelling older people with dementia and their informal primary caregivers.

**Methods:**

Participants with dementia were randomised to an intervention group (*n* = 31, mean age (SD) 78.4 (6.0) years) or usual care (*n* = 30, mean age 79.0 (7.1)). The rehabilitation programme consisted of a 20-week rehabilitation period containing assessments and interventions based on each individual’s goals, and group-based physical exercise plus social interaction twice a week for 16 weeks at a rehabilitation unit. After 5 and 14 months, the interdisciplinary team followed up participants over two four-week periods. For both groups, dates of deaths and decision to move to nursing home over three years, as well as interventions for the relevant periods, were collected. Blinded assessors measured physical functions, physical activity, activities of daily living, cognitive functions, nutritional status, and neuropsychiatric symptoms at baseline and at 5, 12, 24, and 36 months.

**Results:**

Participants in the intervention group received a mean of 70.7 (20.1) interventions during the 20-week rehabilitation period, delivered by all ten team professions. The corresponding figures for the control group were 5.8 (5.9). In the intervention group, all but one participated in rehabilitation planning, including goal setting, and attendance in the exercise and social interaction groups was 74.8%. None of the adverse events (*n* = 19) led to any manifest injury or disease. Cox proportional hazard regression showed a non-significant lower relative risk (HR = 0.620, 95% CI 0.27–1.44) in favour of the intervention for moving to nursing home or mortality during the 36-month follow-up period. Linear mixed-effect models showed non-significant but potentially clinically meaningful between-group differences in gait, physical activity, and neuropsychological symptoms in favour of the intervention.

**Conclusions:**

The rehabilitation programme seems feasible among community-dwelling older people with dementia. The overall results merit proceeding to a future definitive randomised controlled trial, exploring effects and cost-effectiveness. One could consider to conduct the programme earlier in the course of dementia, adding cognitive training and a control attention activity.

**Trial registration:**

The study protocol, ISRCTN59155421, was registered online 4/11/2015.

**Supplementary Information:**

The online version contains supplementary material available at 10.1186/s12877-024-05372-9.

Dementia is common among older people and is the leading cause of dependency in activities of daily living (ADLs) in this population [1]. As the prevalence of dementia will increase and further challenge society’s resources, the World Health Organisation (WHO) recognises dementia as a public health priority [1], and advocates rehabilitation, which encompasses interventions to optimise functioning and reduce disability in individuals’ contexts, as a fundamental health service required to meet the needs of those affected with dementia [2]. However, in contrast to people with other diseases engaging the central nervous system, e.g. multiple sclerosis [3] and stroke [4], rehabilitation is not routinely available in clinical settings for people with dementia [5, 6]. Reasons for this situation, in addition to limited financial resources, may include the challenges involved in managing the complex consequences of dementia, as well as negative attitudes among staff regarding the ability of people with dementia to participate in rehabilitation [7, 8].

People with dementia can experience a good quality of life [9], despite many potentially adverse consequences that may compromise health and well-being. In addition to impaired cognitive function, a gradual decline in walking and balance is common [10, 11]. The reduced cognitive and functional abilities contribute to an increased risk of falls and fractures [12, 13], and may negatively influence levels of daily physical activity [14, 15] and participation in society [11]. Other common consequences that can negatively impact health and complicate care are malnutrition [16], impaired oral health [17], depression [18], neuropsychiatric problems [19], increased risk of complications from diseases [20, 21], and adverse drug reactions [22]. To meet the complex needs of people with dementia, the WHO recommends that multi-professional assessments and interventions are offered; i.e., similar to the recommendations for other neurological conditions [23].

Conducting interventions in the population with dementia can be challenging due to various consequences related to the condition [24]. These challenges include a limited awareness of difficulties in everyday life and anxiety participating in new situations [25], as well as a risk of limited adherence due to reduced memory and executive functions. As the symptoms and course vary considerably between different dementia types, as well as between individuals [1], a person-centred approach, which engages the person as an active partner in the rehabilitation planning [26], appears to be significant in the care of people with dementia [27]. In addition, it seems important to involve the informal primary caregivers in the rehabilitation, as they often provide essential support in enabling everyday life for the person with dementia [1]. Successful rehabilitation could reduce the negative consequences of dementia, increase well-being, and improve opportunities for the person with dementia to remain living in the community. However, there is limited knowledge regarding the feasibility and effects of rehabilitation interventions provided by comprehensive interdisciplinary teams [28] in outpatient settings [29, 30]. Increased knowledge in the area is important for designing future initiatives in dementia care.

The Multidimensional Interdisciplinary Rehabilitation in Dementia (MIDRED) study is a randomised controlled pilot trial (pilot RCT) evaluating the feasibility of a person-centred rehabilitation programme for community-dwelling older people with dementia, including education and support to informal primary caregivers. Experiences of the programme by the participants with dementia and the team staff have been described earlier [31, 32]. The aim of the present study was to further evaluate the feasibility of the rehabilitation programme among participants with dementia; i.e., to explore if a definitive RCT can be done, should be done and, if so, how. Specifically, the study aimed to evaluate participation in rehabilitation planning, delivery of assessments and interventions, adherence to exercise and social gatherings at a day rehabilitation unit, adverse events, retention by estimating blinded assessments and follow-up rates over 36 months, and potential short- and long-term effects.

## Methods

### Setting and participants

The MIDRED study was conducted in Umeå, Sweden. The study protocol was registered 4/11/2015, ISRCTN59155421, at 10.1186/ISRCTN59155421 before the enrolment of the first participant. People with dementia and their informal primary caregivers were recruited from November 2015 to January 2016 at five local health centres in Umeå and the outpatient unit of the Geriatric Centre at the University Hospital in Umeå. Inclusion criteria were dementia according to the International Statistical Classification of Diseases and Related Health Problems (ICD) 10th revision, 60 years and older, living in the community, ability to rise from a chair with armrests with help from no more than one person, and ability to hear and understand spoken Swedish sufficiently to participate in assessments. Criteria also included a Mini-Mental State Examination (MMSE) [33] score of 10 or higher, no initiated move to a nursing home [34], including respite care, expected survival of more than six months, and approval from the participants’ physician to participate in the study. Based on given information about the study, staff at the six different participating units identified potential participants through lists of people with dementia collected from medical records. If the individual accepted contact with project staff, they received written information by post, and if approved, a home visit was made to give additional written and oral information about the study and to assess whether the potential participant met the inclusion criteria. A maximum of two informal primary caregivers (spouse, child, or other relative or friend involved in the care of the person with dementia) of each participant with dementia were also invited to participate in the study after they were given oral and written information.

## Sample size and randomisation

Based on a power analysis of the proportion of people with dementia continuing to live in the community at the 24-month follow-up assessment, the original RCT was planned to include 179 participants with dementia. Due to lack of funding, only the first of three pre-planned groups were included in the trial. Therefore, the study is reported as a pilot trial, evaluating feasibility of the rehabilitation programme [35]. Participants with dementia, together with their informal primary caregivers, were randomised with a 1:1 allocation, to a control group (usual care) or a person-centred multidimensional rehabilitation programme for the person with dementia, including education and support for the informal primary caregiver. In order to prevent a random oblique distribution of participants´ characteristics between the groups, the participants were stratified by type of dementia (Dementia with Lewy bodies or Parkinson´s disease with dementia; vascular dementia; Alzheimer´s disease; mixed Alzheimer´s disease and vascular dementia or unspecified dementia) and household living conditions (living alone or with a partner/child), thus 4*2 strata (four types of dementia and two living conditions). Within the strata, they were ranked according to MMSE score. If the MMSE score was equal between participants, they were ranked according to age and then sex (female sex first). Using a die, two persons not involved in the study performed the randomisation with a block size of two from each stratum to balance intervention/control. Randomisation was performed after the inclusion process and the baseline assessments to eliminate the possibilities of selection bias.

### The rehabilitation programme

The rehabilitation programme, starting in February 2016, consisted of assessments and interventions provided by the team staff over a 20-week rehabilitation period and two follow-up periods of four weeks each after 5 and 14 months. Professions involved in the team, working full time, included one assistant nurse, two occupational therapists, two physiotherapists (PT), and one social worker. In addition, one clinical pharmacist, one dental hygienist, one dietician, one neuropsychologist, one nurse, and two physicians, worked part-time in the intervention. The team practiced person-centred care [26] and all staff had experience working with people with dementia. Before the intervention, team staff took part in a compilation of common problem areas related to dementia including possible interventions for each area, based on current literature, as well as routines and schedules for implementing the rehabilitation programme. During the initial four weeks, the team staff identified problems and needs, as well as resources, within ten potential intervention areas for the person with dementia: functional capacity; cognitive function; ADL performance; falls; participation in society; physical activity; nutritional status; medical conditions including oral health; neuropsychiatric symptoms; and pharmacological treatment. Intervention needs were defined based on the findings. The social worker assessed the need for individual support and counselling for the informal primary caregiver through interviews. The results from the baseline assessments were available to team staff as complementary information. At a separately scheduled meeting, each participant with dementia, together with his/her informal primary caregiver(s), when present, and encouraged by two familiar staff representatives, formulated their own rehabilitation goals in any area. Staff representatives guided the participant, when needed, by referring to important areas of intervention that emerged during the assessments. Individual goals, interventions and continuous follow-up meetings to evaluate goal fulfilment were documented in a rehabilitation plan, consented by the participant. The rehabilitation plan, photos of the staff members, a schedule of activities and other relevant information, were collected in a folder kept by the participant. Based on the goals, relevant professionals formed a rehabilitation team for each participant. The progress of the interventions and any new problems were discussed and evaluated in weekly meetings that included all team staff. The team staff followed the participants’ body weight regularly and performed reassessments when needed. In the end of the rehabilitation period, the dental hygienist followed up all participants. Participating informal primary caregivers were offered individual support and counselling up to six occasions by the social worker, and other professionals could be engaged if needed. Informal primary caregivers were also invited to participate in group-sessions offering education and support, led by the nurse and the social worker, to improve self-management skills.

Participants with dementia were offered interventions based on the individual´s goals, performed by relevant professions in the team, and physical exercise with social gatherings in small groups twice a week for 16 weeks at a day rehabilitation unit. Interventions were conducted at the day rehabilitation unit at the Geriatric Centre, in the homes of the participants with dementia and/or in the community.

Physical exercises were individualised and conducted in groups of three or four participants, supervised by two PT, twice a week for approximately 45 min. The assistant nurse was responsible for organising transportation to the day rehabilitation unit for all participants. In addition, the assistant nurse made phone calls to remind participants who lived alone and would benefit from the support. The assistant nurse and other available staff assisted participants in arriving and leaving the clinic and organised social gatherings during coffee breaks after the exercise sessions, ensuring that participants felt comfortable and welcomed. The exercises were based on the High-Intensity Functional Exercise Program (HIFE) [36]. Exercises were selected based on an individual´s degree of functional ability and limitations, and progressed during the period with respect to participants´ health, functional status, cognitive ability, and neuropsychiatric symptoms. The participants were individually supervised to achieve the highest possible exercise intensity while ensuring their safety. The PT obtained updates on the participant´s health status and could consult the physician or the nurse when needed. When possible, participants were offered individual exercise at home when they were not able to participate in the group session. Participants with dementia also received individual recommendations and guidance for the achievement of recommended physical activity levels [37], including support in attending training groups or gyms, and applying for assistance in exercising or walking by staff from social services at home. Written information on different physical and social activities available in local society was distributed, according to suitability and participants’ interest.

At five and 14 months after the 20-week rehabilitation period, follow-up periods of four weeks each were performed, in which participants were reassessed by team staff following the same routine as in the 20-week rehabilitation period. The interventions and activities initiated or proposed during the rehabilitation period were followed up and complementary interventions were offered, if needed. At the end of the 20-week period and the two follow-up periods, the participant received written summaries from the follow-up meetings, including performed interventions and goal fulfilment, as well as advice and encouragement on maintaining their activities.

### Control

Participants randomised to the control group received usual care; i.e., they continued their ordinary health care contacts according to their needs and planned follow-ups in outpatient settings. All participants were registered at a local health centre, which had the primary responsibility for health care, but could also be followed up with regard to dementia symptoms by specialists at the outpatient unit of the Geriatric Centre at the University Hospital in Umeå, if needed. The study imposed no restrictions on participants concerning rehabilitative efforts, by for example PT or occupational therapist.

### Measurements

During home visits, trained PTs, blinded to allocation and previous test results, performed structured assessments with the participants with dementia, and oral questionnaires with relatives or, when required, with care staff, at baseline, and 5, 12, 24, and 36 months. Data on participants´ medical history and current pharmacological treatment were collected by reviewing electronic medical records, in addition to the questionnaires. A specialist in geriatric medicine (YG) reviewed all test protocols at the follow-ups, to assess whether any participant (control and intervention) had any medically serious condition; e.g., severe depression or malnutrition. In those cases, their next of kin were informed and offered advice how to get help. If applicable, nurse or nursing staff were informed.

### Blinding procedure

A strategy was developed in advance to preserve the assessors´ blinding to group allocation. In the end of the 20-week rehabilitation period, team staff asked the participant and the informal primary caregiver in the intervention group to remove the folder and other equipment that may reveal group affiliation. An individual other than the assessor made the appointment for home visit testing and encouraged the participant with dementia and their informal caregiver not to reveal group affiliation. During assessment, the assessor was instructed to not talk about what have happened before or what would happen in the future and focused the conversation on the ‘here and now’. If the blinding was broken anyway, the assessor was replaced, according to a pre-planned schedule, to preserve blindness.

## Outcome measures

The proportions of people with dementia who were living in the community (the inverse of death or institutionalisation combined) at the 24- and 36-month follow-up assessments were collected through a review of medical records, and through dates decisions were made to move to a nursing home [34] which were provided by the social authorities of the municipality. Global cognitive function was assessed using the MMSE [33] and executive function was assessed using the Verbal fluency test [38] where participants were asked to name as many animals as possible within one minute. Balance was assessed with the Berg balance scale (BBS) [39, 40]. Functional leg muscle strength was assessed with 30-second Chair stand test [41] using a standard chair without armrests. Gait speed with the habitual walking aid was assessed by a 2.4-metres timed test [42] starting in standing position, twice at usual and maximum speed, respectively, and once backwards in their usual pace. The same walking aid was used on all test occasions, if any at baseline. The participants´ physical activity levels of the previous week were measured using the International Physical Activity Questionnaire adapted for adults aged ≥ 80 years (IPAQ-E 80+) [43] and calculated as total physical inactivity and total physical activity time. The informal primary caregiver or care staff confirmed the data. To avoid an impact of the intervention on the IPAQ-E 80 + assessment, the 5-month follow-up assessment was conducted at least seven days after the end of 20-week rehabilitation period. Dependence in ADLs was assessed by questioning the informal caregiver or care staff by using the Lawton and Brody scales containing six P-ADL and eight I-ADL domains [44, 45] and the motor domain of Functional Independence Measure (FIM) [46, 47]. Nutritional status according to Mini Nutritional Assessment (MNA, 0–30) [48] including body mass index (kg/m^2^), was assessed. Neuropsychiatric symptoms were assessed by questioning the informal primary caregiver or care staff, using the Neuropsychiatric Inventory, (NPI) [49]. Both total score and subscales were analysed [50].

### Delivery of assessments and interventions, attendance, exercise intensity, and adverse events

For the participants in the intervention group, every assessment and intervention provided by the team staff was documented in medical records. After the exercise session, the PT completed a structured report for each participant, including reasons for non-attendance, estimated exercise intensity [51], reasons for not achieving high intensity, and adverse events. In addition, adverse events that occurred during the visit at the day rehabilitation unit during the 20-week rehabilitation period and the two follow-ups were recorded. One specialist in geriatric medicine (YG), one nurse (BO), and one PT (AT) assessed the severity of the adverse events in consensus [51]. The specialist in geriatric medicine (YG) evaluated whether the deaths of participants with dementia in the intervention group were related to the intervention. For participants in the intervention and the control groups, data on all medical, dental, and rehabilitative assessments and interventions, performed in any outpatient settings during the 20-week rehabilitation period and the two follow-up periods, were collected and coded from medical and dental care records [52].

### Baseline and descriptive measurements

Depressive symptoms were screened using the Geriatric Depression Scale, the 15-item version (GDS-15) [53]. The Philadelphia Geriatric Center Morale Scale (PGCMS) [54] was used to assess psychological well-being. Self-perceived health was assessed using the first question of the Medical Outcome Study 36 item Short-Form Health Survey [55]. After the 36-month follow-up, an experienced specialist in geriatric medicine (YG) reviewed all medical diagnoses. Dementia and depressive disorders were diagnosed according to the Diagnostic and Statistical Manual of Mental Disorders, fourth edition, text revision (DSM-IV, TR) [56] and the dementia diagnoses were verified using information from participants’ medical records, prescriptions, and assessments.

### Statistical analysis

All analyses were based on the intention-to-treat principle using all available data at each time-point on each participant according to their original group assignment, regardless of attendance in the intervention. No imputation of missing values was performed to be able to provide values for dispersion. Differences between the intervention and control group in baseline characteristics (selected a priori as potential confounders), were calculated using Student’s t-test or the Pearson´s chi-square test (Table 1). According to an a priori strategy, all between-group analyses were adjusted for age, sex, and any imbalances between groups at baseline (*p* < 0.05); in this case chronic lung disease, *p* = 0.04.

**Table 1:** Characteristics of the participants and baseline measures

Baseline characteristics	Total *n* = 60	Intervention, *n* = 31	Control, *n* = 29	*p*-value
*Age*,* years (SD)*	78.9 (6.4)	78.5 (6.1)	79.5 (6.7)	0.537
*Female*,* n (%)*	35 (58.3)	20 (64.5)	15 (51.7)	0.315
*Social service at home or remunerated help with P-ADLs from informal caregiver n (%)*	22 (36.7)	12 (38.7)	10 (34.5)	0.734
*- Hours per week*,* mean (SD)*	2.6 (5.3)	2.0 (3.15)	3.3 (7.0)	0.345
*Lives alone*,* n (%)*	21 (36.7)	10 (32.3)	11 (37.9)	0.464
*Education*,* year at school*,* mean (SD)*	11.0 (3.8)	11.6 (3.7)	10.3 (3.8)	0.208
***Dementia type***,*** n (%)***				
- *Vascular*	11 (18.3)	5 (16.1)	6 (20.7)	
- *Alzheimer’s disease (AD)*	29 (48.3)	16 (51.6)	13 (44.8)	
- *Mixed (AD + vascular)*	11 (18.3)	5 (16.1)	6 (20.7)	
- *Dementia with Lewy Bodies (DLB)*, *Parkinson’s disease with dementia and mixed (DLB + vascular)*	9 (15.0)	5 (16.1)	4 (13.7)	0.537
***Diagnoses (%)***				
- *Depressive disorders*	29 (48.3)	16 (51.6)	13 (44.8)	0.599
- *Malignancy last 5 years*	25 (41.7)	13 (41.9)	12 (41.4)	0.965
- *Chronic lung disease*	13 (23.3)	10 (32.3)	3 (10.3)	0.040
- *Previous stroke*	11 (18.3)	5 (16.1)	6 (20.7)	0.648
- *Previous myocardial infarction*	7 (11.7)	4 (12.9)	3 (10.3)	1.000
- *Heart failure*	12 (20.0)	6 (19.4)	6 (20.7)	0.897
- *Angina pectoris*	10 (16.7)	4 (12.9)	6 (20.7)	0.419
- *Diabetes*	9 (15.0)	4 (12,9)	5 (17.2)	0,727
- *Previous hip fracture*	4 (6.7)	2 (6.5)	2 (6.9)	1.000
- *Osteoarthritis*,* lower extremity*	24 (40.0)	13 (41.9)	11 (37.9)	0.833
***Prescribed medication for regular use***, ***n (%)***				
*Analgesics*, *N02A*, *N02B*	9 (15.0)	4 (12.9)	5 (17.2)	0.727
*Antidementia drugs*				
* - Cholinesterase inhibitor*,* N06DA*	43 (71.7)	21 (67.7)	22 (75.9)	0.485
* - Memantine*,* N06DX01*	11 (18.3)	6 (19.4)	5 (17.2)	0.833
*Antidepressants*,* N06A*	19 (31.7)	9 (29.0)	10 (34.5)	0.650
*Antipsychotics*,* N05A*	4 (6.7)	4 (12.9)	0 (0)	0.113
*Benzodiazepines*,* N05BBA*	5 (8.3)	3 (9.7)	2 (6.9)	1.000
*Diuretics*,* C03*	14 (23.3)	8 (25.8)	6 (20.7)	0.640
*Vitamin D-Ca supplement*,* A11C05*,* A12AX*	9 (15.0)	3 (9.7)	6 (20.7)	0.465
*Drugs with anticholinergic properties* [57]	41 (67.2)	20 (64.5)	20 (69.0)	0.715
*Number of medications mean* (*SD)*,* range*	6.5 (3.1), 1–15	6.4 (3.2), 1–14	6.6 (3.1), 1–15	0.836
***Assessments***				
- *Vision; can read 5 mm capital letters*,* with or without glasses*,* n (%)*	59 (98.3)	31 (100)	28 (96.6)	0.483
- *Hearing; can hear a conversation at normal voice level*,* with or without hearing aids*,* n (%)*	58 (96.7)	31 (100)	27 (93.1)	0.229
- *BMI*,* mean (SD)*,* range*	26.0 (4.4), 18.5–37.5	26.1 (4.7), 19.6–36.2	25.8 (4.2), 18.5–37.5	0.766
- *Self-reported health*,* good*,* very good or excellent*,* n (%)*	44 (73.3)	24 (77.4)	20 (69.0)	0.459
- *GDS*,* 0–15*,* mean (SD)*,* range*	3.4 (2.7), 0–11	3.7 (3.0), 0–11	3.2 (2.4), 0–9	0.453
- *PGCMS*,* 0–17*,* mean (SD)*,* range*	12.4 (3.0), 5–17	12.2 (3.4), 5–17	12.7 (2.7), 6–17	0.559

*SD *Standard deviation, *GDS *Geriatric Depression Scale, *PGCMS *Philadelphia Geriatric Center Morale Scale

Survival analysis was performed using Cox proportional hazard regression, with events defined as moving to a nursing home or death, whichever came first over the three-year follow-up, and adjusted for age, sex, and chronic lung disease. The proportionality of hazards was statistically tested using Schoenfeld´s residuals. Longitudinal changes in the other outcomes from baseline to 5, 12, 24, and 36 months were analysed using linear mixed-effect models with interaction terms for group and time-point and adjustment for age, sex, and chronic lung disease as fixed effects and individual as random effects. The baseline value of the outcome was included as the first time-point in the dependent variable in order to use all available data in the analyses. The SPSS software version 26.0 (IBM SPSS Inc., Chicago, IL) was used for all data analyses. All analyses were two-tailed, and *p* < 0.05 was considered to indicate statistical significance.

## Results

### Participants

**Fig. 1:**
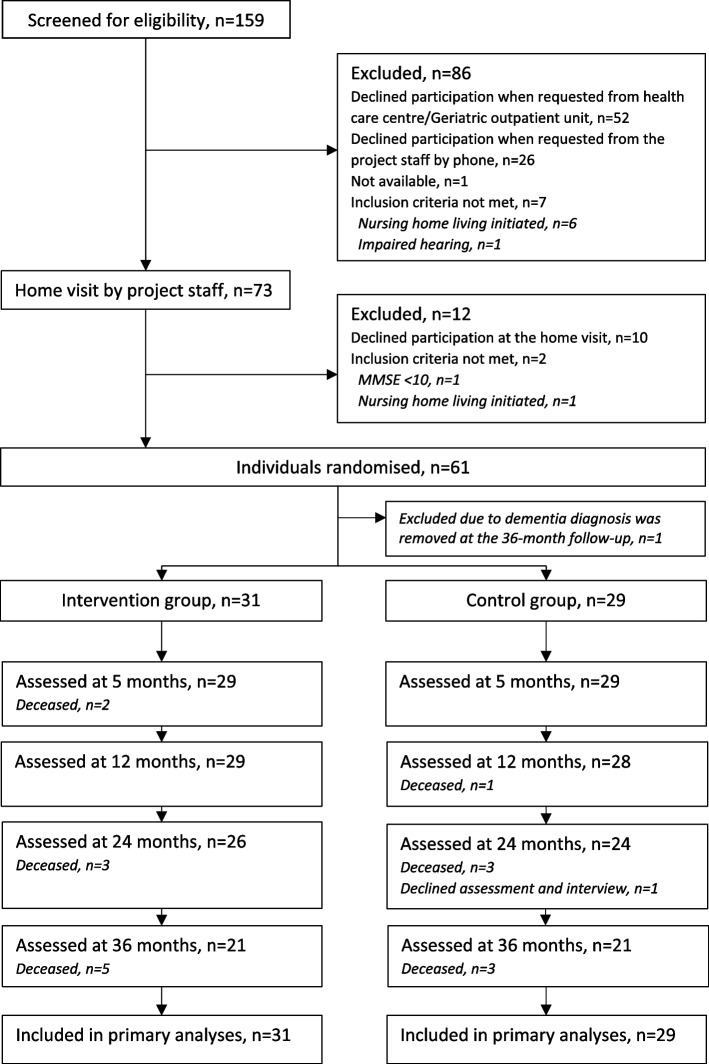
Flow of participants with dementia through the study. Assessed = Participated in assessment and/or informal primary caregiver or care staff was interviewed. MMSE = Mini-Mental State Examination

Of 159 persons screened for eligibility, 73 agreed to a home visit by project staff and of those, 61 persons with dementia, together with 67 informal primary caregivers, were randomised (Fig. 1). One participant with dementia and the associated informal primary caregiver were excluded from the statistical analysis since it emerged, from medical records, that the Alzheimer´s disease diagnosis had been removed at the 36-month follow-up. Thus, 31 participants in the intervention group and 29 in the control group were included in the final analyses. Sex did not differ (*p* = 0.554) between participants included in the study and those who declined, but those who declined were older (mean age 78.9 years versus 82.2 years, *p* = 0.007).

The baseline characteristics of the participants in the intervention and control group are presented in Table 1. In total, 35 participants (58.3%) were women, and the mean (SD) MMSE score was 20.9 (3.9). Twelve participants, equally divided between the intervention and control group, had severe cognitive impairment (MMSE < 18). Of the 60 participants, 21 lived alone, and 57 of the participants had one or two informal primary caregivers participating in the study.

### Participation in rehabilitation planning

All participants, except one (the spouse denied the participant with dementia participation in the 20-week rehabilitation period), were active in establishing the rehabilitation plan, including formulating goals and planning the interventions with staff representatives.

### Assessments and interventions delivered to the intervention and control groups

In total, the participants with dementia in the intervention group received a mean (SD) of 29.6 (6.7) different assessments and 70.7 (20.1) interventions in outpatient settings during the 20-week rehabilitation period. The team staff provided 88.0% and 92.5% of the total health and dental care assessments and interventions, respectively. Corresponding figures for the control group were 4.9 (6.7) assessments and 5.8 (5.9) interventions; where physicians and nurses provided 48.4% of assessments and 49.1% of interventions, physiotherapists provided 17.6% and 25.5%, respectively, and various health and oral care professions provided the remaining. All participants with dementia in the intervention group received assessments and interventions delivered by the team staff. All ten professions delivered assessments and interventions to various extent (Table 2). For 23 (74.2%) participants, team staff contacted other care providers regarding participants´ needs for social, medical, and dental care interventions. Type of assessments and interventions in the 20-week rehabilitation period, as well as for the two follow-up periods, for each profession in the team, are presented in Table A. 1–20, Additional file 1.

**Table 2:** Team staffs´ assessments, interventions, and contacts with other care providers, during the 20-week rehabilitation period^a^

Profession	Participants receiving assessment *n* (%)	Assessments^b^ per participant assessed median (IQR)	Participants receiving intervention *n* (%)	Interventions^b^ per participant receiving intervention median (IQR)	Contacts with other care providers^c^, number of participants, *n* (%)
Assistant nurse	6 (19.4)	1.5 (1.0–2.25)	1 (3.2)	2.0 (2.0–2.0)	0
Dental hygienist	30 (96.8)	1.0 (1.0–1.0)	29 (93.5)	3.0 (2.0–4.0)	6 (19.4)
Neuropsychologist	7 (22.6)	1.0 (1.0–1.0)	8 (25.8)	2.0 (1.0–4.0)	0
Nurse	4 (12.9)	1.0 (1.0–4.75)	3 (9.7)	1.0 (1.0–1.0)	2 (6.5)
Dietician	30 (96.8)	7.0 (6.75–8.0)	8 (25.8)	2.5 (1.0–3.75)	2 (6.5)
Occupational therapist	31 (100)	6.0 (6.0–7.0)	28 (90.3)	3.0 (2.0–5.0)	14 (45.2)
Clinical pharmacist	30 (96.8)	1.0 (1.0–1.0)	9 (29.0)	1.0 (1.0–1.0)	0
Physiotherapist	30 (96.8)	7.0 (4.0–9.0)	Group exercise: 30 (96.8) Other interventions: 23 (64.5)	Group exercise: 53.5 (41.0–56.5) Other interventions: 2.0 (1.0–4.0)	9 (32.3)
Physician	30 (96.8)	3.0 (3.0–4.0)	16 (51.6)	1.0 (1.0–2.0)	3 (9.7)
Social worker	1 (3.2)	1.0 (1.0–1.0)	16 (51.6)	1.0 (1.0–2.0)	13 (41.9)
**Total**	**31 (100)**	**26.0 (23.0–29.0)**	**31 (100)**	**71.0 (57.0–79.0)**	**23 (74.2)**

*IQR *Interquartile range

^a^Amount of assessments and interventions directed at the participant, and initiatives directed at other care providers, during the 20-week rehabilitation programme for each profession in the team, presented per participant with dementia in the intervention group (*n* = 31)

^b^Each assessment and intervention, documented in medical records, was coded based on the National board of Health´s Classification of care efforts [52]. Each contact with a participant could include more than one assessment/intervention

^c^Contacts initiated by the team with other care providers, regarding the participants´ needs for social and medical care interventions

### Attendance; the intervention group´s visits for exercise and social gathering

The attendance at the day rehabilitation unit was 74.8%, 696 of 930 sessions. In addition, 17 individual exercise sessions were conducted at home or respite care/nursing home (total exercise attendance 76.7%). Reasons for individual sessions included tiredness after illness, respite/nursing home care, and anxiety and sleep disturbances before coming to the day rehabilitation unit. For all exercise sessions, the median (interquartile range) individual exercise attendance rate and effective workout time per session were 90.0% (63.3–93.3%) and 26.6 min (24.5–34.3 min), respectively. The most common reasons for the non-attended exercise sessions (217/930) included illness/hospital care (29.0%), travels and other visits (21.8%), and deceased status (20.3%).

### Exercise intensity

In total, lower-limb strength exercises were performed at high intensity; i.e., 8 to 12 repetition maximum (RM), in 59.0% of the attended sessions, and medium intensity; i.e., 13 to 15 RM, in 27.8% (421 and 198 out of 713 sessions, respectively). Balance exercises were performed at high intensity; i.e., performed at, or near the limit of maintaining an upright position, in 82.6% of the attended sessions, and at medium intensity; i.e., postural stability was not fully challenged, or fully challenged in only a minority of the exercises, in 16.0% (589 and 114 out of 713 sessions, respectively). Pain was the most common reason for medium or low strength and balance exercise intensity (45.9% and 13.0%, respectively), followed by build-up exercise (32.5% and 15.1%, respectively).

### Adverse events

In total, 19 adverse events were recorded. Eighteen adverse events occurred during the 713 exercise sessions (2.5%), among 13 (41.9%) participants. Seventeen of the adverse events were assessed as “minor and temporary”. The other two events were assessed as “serious”; one fall incident during an exercise session and one near fall incident in the dressing room, prevented by staff. None of the events led to any manifest injury or disease. The most common adverse events were musculoskeletal (soreness, pain) 9/19 (47.4%). Other adverse events included; psychological, dizziness, falls, near fall incident, cardiorespiratory, tiredness, and tripping (hurting the foot). No deaths were related to the intervention.

### Blinded assessments and follow-up rates for the intervention and the control groups

The consecutive follow-up assessments were all accomplished with preserved blinding. On four occasions, the group affiliation was revealed, and the assessor was replaced. In the intervention group, the follow-up rates for participation in assessments and/or questioning informal primary caregivers or care staff at 5, 12, 24, and 36 months were 93.5%, 93.5%, 83.9%, and 67.7%, respectively (Fig. 1). Of those participants still alive, all performed the majority of the assessments (Tables 3 and 4), and all oral questionnaires with informal primary caregivers or care staff were completed at the follow-ups. The corresponding follow-up rates in the control group were 100%, 96.6%, 82.8%, and 72.4% (Fig. 1). Of those still alive, one participant declined to participate in the assessments at 12 months, and three and two participants declined at 24 and 36 months, respectively. All oral questionnaires were completed, except for a single informal primary caregiver who declined at 24 and 36 months.

### Outcomes

**Fig. 2:**
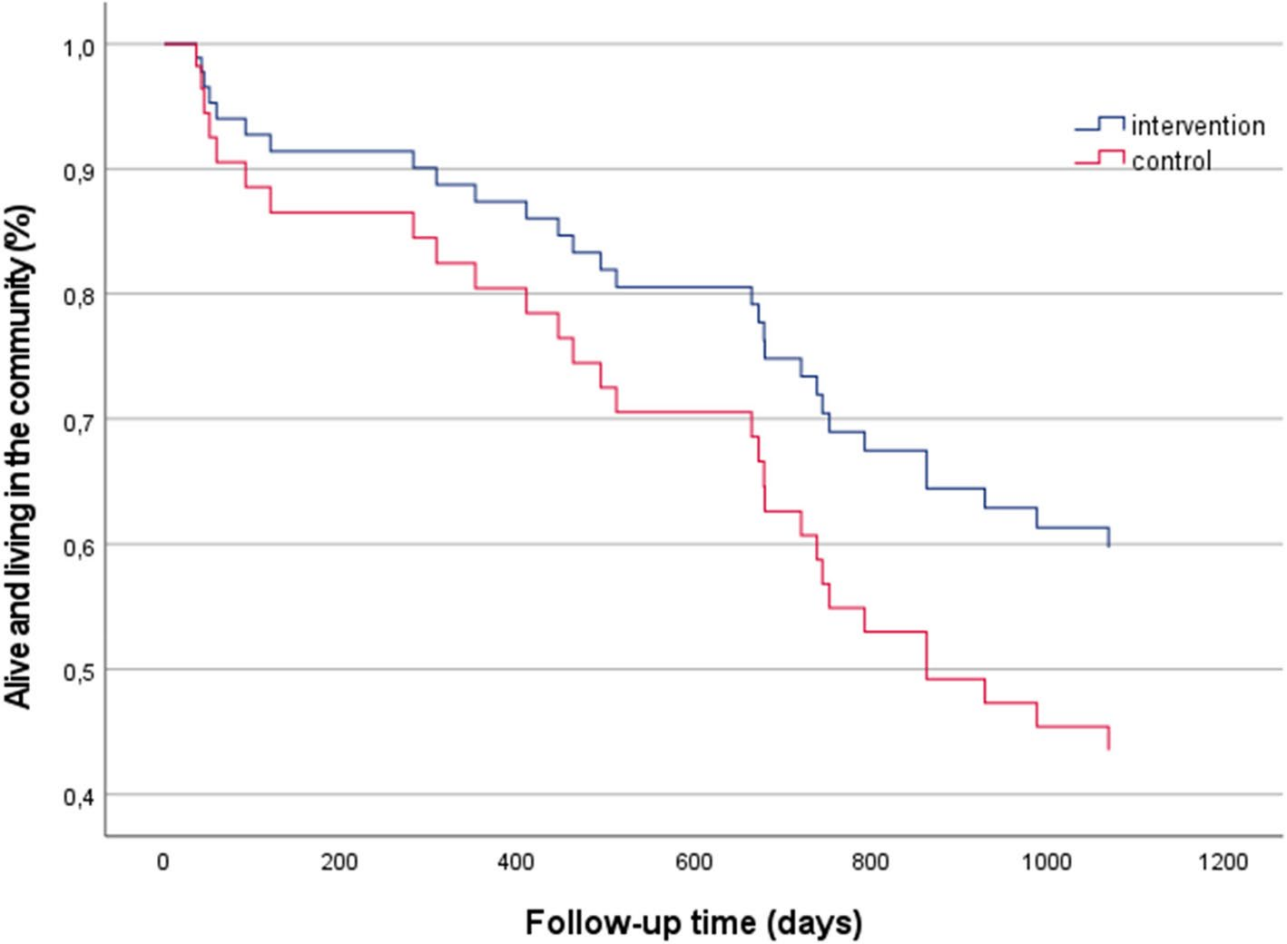
Survival curve, based on Cox proportional hazard regression model, with event defined as death or move to nursing home (whichever came first), adjusted for age, sex, and chronic lung disease, for the intervention group and the control group from baseline to three years’ follow-up

After 24 months, 19 (61.3%) participants in the intervention group and 21 (72.4%) participants in the control group were alive and lived in the community. Nine and seven participants had moved to a nursing home, respectively. After 36 months, 17 (54.8%) participants in the intervention group and 14 (48.3%) participants in the control group were alive and lived in the community. Nine and twelve participants had moved to nursing homes, respectively. When using the Cox proportional hazards regression analysis to compare time to moving to nursing home or mortality, the intervention group had a hazard ratio of 0.62, 95% CI 0.27–1.44, *p* = 0.265, relative to the control group, during the three-year follow-up period (Fig. 2).

There were no statistically significant between-group effects in the outcomes related to physical function, physical activity, ADL, cognitive function, nutrition, and neuropsychiatric symptoms (Tables 3 and 4). The between-group difference for maximal and usual gait speed (m/s, 95% CI) was 0.142, -0.03–0.31, *p* = 0.096 at 5 months, and 0.086, -0.01–0.18, *p* = 0.082 at 12 months), respectively, favouring the intervention group. Although wide confidence intervals, the between-group differences remained during the follow-ups, favouring the intervention group. Similar; i.e., between-group differences over 36 months with wide confidence interval favouring the intervention group, were seen in the areas of physical activity and inactivity, as well as neuropsychiatric symptoms (Tables 3 and 4).

**Table 3:** Within- and between-group differences from baseline in measures of physical function and physical activity

	Baseline value and within-group difference^a^				Between-group difference	
Measure	n	Intervention, mean (SE)	n	Control, mean (SE)	Mean (95% Confidence Interval)	p-value
**Physical function**						
** The Berg balance scale**,** 0–56 p**						
* Baseline value*	*31*	*46.61 (1.97)*	*29*	*48.35 (2.14)*		
5 months	29	1.02 (1.74)	29	-1.03 (1.76)	2.05 (-2.83–6.93)	0.408
12 months	29	-2.09 (1.74)	27	-2.62 (1.80)	0.53 (-4.42–5.47)	0.833
24 months	26	-7.38 (1.81)	22	-6.62 (1.93)	-0.76 (-5.97–4.46)	0.775
36 months	19	-9.63 (2.01)	20	-9.26 (1.99)	-0.37 (-5.95–5.20)	0.896
**Usual gait speed**, **m/s**, **mean value of two trials**						
* Baseline value*	*31*	*0.691 (0.04)*	*29*	*0.703 (0.05)*		
5 months	29	0.045 (0.03)	28	0.002 (0.04)	0.043 (-0.05–0.14)	0.375
12 months	28	-0.015 (0.03)	27	-0.101 (0.04)	0.086 (-0.01–0.18)	0.082
24 months	24	-0.036 (0.04)	22	-0.112 (0.04)	0.077 (-0.03–0.18)	0.145
36 months	19	-0.109 (0.04)	18	-0.135 (0.04)	0.026 (-0.09–0.14)	0.644
**Maximal gait speed**, **m/s**, **best value of two trials**						
* Baseline value*	*31*	*1.033 (0.06)*	*29*	*1.088 (0.08)*		
5 months	29	0.147 (0.06)	28	0.005 (0.06)	0.142 (-0.03–0.31)	0.096
12 months	28	0.013 (0.06)	27	-0.025 (0.06)	0.038 (-0.13–0.21)	0.658
24 months	24	-0.159 (0.06)	22	-0.238 (0.07)	0.079 (-0.10–0.26)	0.386
36 months	18	-0.119 (0.07)	18	-0.194 (0.07)	0.075 (-0.12–0.27)	0.448
**Backwards gait speed**, **m/s**						
* Baseline value*	*31*	*0.373 (0.03)*	*29*	*0.402 (0.03)*		
5 months	29	0.028 (0.04)	28	-0.044 (0.04)	0.072 (-0.05–0.19)	0.230
12 months	27	-0.036 (0.04)	27	-0.046 (0.04)	0.010 (-0.11–0.13)	0.872
24 months	23	-0.029 (0.04)	21	-0.062 (0.05)	0.033 (-0.09–0.16)	0.609
36 months	18	-0.056 (0.05)	18	-0.121 (0.05)	0.065 (-0.07–0.20)	0.340
**Chair stand 30 s**, **number of raises from a chair in 30 s**						
* Baseline value*	*31*	*10.30 (1.02)*	*29*	*9.73 (1.09)*		
5 months	29	-0.24 (1.43)	29	0.21 (1.46)	-0.45 (-4.48–3.58)	0.826
12 months	29	-1.00 (1.43)	27	-1.21 (1.48)	0.21 (-3.86–4.27)	0.920
24 months	26	-1.24 (1.48)	22	-0.81 (1.57)	-0.43 (-4.67–3.82)	0.844
36 months	19	-1.77 (1.62)	20	-2.70 (1.61)	0.93 (-3.57–5.43)	0.684
**Physical activity**						
** IPAQ-E 80 +** ^b^; **total inactivity time per 24 h (hours)**						
* Baseline value*	*31*	*19.40 (0.64)*	*29*	*19.73 (0.68)*		
5 months	29	-1.52 (0.90)	29	-0.11 (0.91)	-1.407 (-3.93–1.11)	0.273
12 months	28	-0.49 (0.91)	28	0.16 (0.92)	-0.649 (-3.19–1.89)	0.615
24 months	26	-0.82 (0.93)	24	-0.14 (0.96)	-0.68 (-3.31–1.94)	0.609
36 months	21	-0.24 (0.98)	21	0.27 (1.00)	-0.51 (-3.26–2.25)	0.718
**IPAQ-E 80 +** ^**c**^; **total physical activity time per 24 h (minutes)**						
* Baseline value*	*31*	*28.9 (9.25)*	*29*	*45.0 (9.93)*		
5 months	29	35.2 (13.1)	29	6.5 (13.3)	28.6 (-8.0–65.3)	0.125
12 months	29	2.8 (13.1)	28	-17.7 (13.4)	20.5 (-16.3–57.3)	0.275
24 months	26	-0.7 (13.5)	24	-19.7 (13.9)	19.0 (-19.1–57.1)	0.327
36 months	21	5.6 (14.3)	21	-14.1 (14.5)	19.7 (-20.3–59.7)	0.334

Linear mixed effect models adjusted for age, sex, and chronic lung disease were used to analyse within- and between-group differences

*SE *Standard error

^a^Change from the baseline value.

^b,c^The International Physical Activity Questionnaire adapted for adults aged ≥80 years (IPAQ-E 80+)

^b^total inactivity time=time spent lying down for sleep at night, lying down for rest during the day, and sitting in hours/day

^c^total physical activity time=time spent walking and/or performing physical activity with moderate and/or vigorous effort (in ≥10 minute bouts) in minutes/day. Higher values represents better performance, except for IPAQ-E 80+; total inactivity time

**Table 4:** Within- and between-group differences from baseline in measures of ADLs, nutrition, cognition, and neuropsychiatric symptoms

	Baseline value and within-group difference^a^				Between-group difference	
Measure	n	Intervention mean (SE)	n	Control mean (SE)	Mean (95% Confidence Interval	p-value
**Activities of daily living**						
** Lawton and Brody scale (P- and I-ADLs)**,** 14–61**						
* Baseline value*	*31*	*27.80 (1.75)*	*29*	*26.64 (1.88)*		
5 months	29	0.29 (2.47)	29	0.10 (2.51)	0.19 (-6.73–7.11)	0.958
12 months	29	3.46 (2.47)	28	3.19 (2.53)	0.27 (-6.68–7.22)	0.939
24 months	26	6.23 (2.54)	24	5.00 (2.63)	1.23 (-5.98–8.43)	0.738
36 months	21	9.06 (2.70)	21	7.71 (2.73)	1.35 (-6.21–8.92)	0.725
**Functional Independent Measure**, **13–91**						
* Baseline value*	*31*	*82.39 (2.81)*	*29*	*82.81 (3.01)*		
5 months	29	-2.19 (3.96)	29	-1.90 (4.02)	-0.29 (11.41–10.82)	0.959
12 months	29	-5.50 (3.96)	28	-3.61 (4.06)	-1.89 (-13.05–9.27)	0.739
24 months	26	-9.47 (4.08)	24	-4.87 (4.23)	-4.60 (-16.17–6.97)	0.434
36 months	21	-11.68 (4.34)	21	-10.88 (4.39)	-0.80 (-12.94–11.35)	0.897
**Nutrition**						
** Mini Nutrition Assessment**,** 0–30**						
* Baseline value*	*31*	*23.41 (0.60)*	*29*	*23.22 (0.64)*		
5 months	29	0.35 (0.84)	29	-0.38 (0.86)	0.73 (-1.64–3.10)	0.545
12 months	29	0.38 (0.84)	27	0.10 (0.87)	0.28 (-2.11–2.67)	0.815
24 months	26	-1.35 (0.87)	22	-1.40 (0.92)	0.05 (-2.45–2.54)	0.970
36 months	21	-1.11 (0.92)	20	-1.54 (0.95)	0.43 (-2.17– 3.04)	0.744
**Cognitive function**						
** Mini-Mental State Examination**,** 0–30**						
* Baseline value*	*31*	*20.71 (1.03)*	*29*	*20.75 (1.11)*		
5 months	29	-0.29 (1.46)	29	-0.45 (1.48)	0.16 (-3.94–4.26)	0.939
12 months	29	-2.15 (1.46)	27	-1.56 (1.51)	-0.59 (-4.72–3.55)	0.780
24 months	26	-2.38 (1.50)	22	-1.28 (1.60)	-1.10 (-5.42–3.22)	0.617
36 months	21	-3.58 (1.60)	20	-3.52 (1.64)	-0.06 (-4.57–4.45)	0.979
**Verbal fluency test**, **number of animals listed in one minute**						
* Baseline value*	*31*	*11.49 (0.98)*	*29*	*10.93 (1.05)*		
5 months	29	0.38 (1.38)	29	-0.41 (1.41)	0.79 (-3.09–4.68)	0.688
12 months	28	-1.08 (1.40)	27	-0.19 (1.43)	-0.88 (-4.82–3.06)	0.660
24 months	25	-1.33 (1.44)	21	-1.18 (1.53)	-0.14 (-4.29–4.00)	0.946
36 months	20	-2.16 (1.54)	20	-3.41 (1.56)	1.25 (-3.06–5.55)	0.568
**Neuropsychiatric symptoms**						
** Neuropsychiatric Inventory**,** sum score**,** 0–144**						
* Baseline value*	*31*	*9.71 (2.19)*	*29*	*8.66 (2.35)*		
5 months	29	0.46 (3.09)	29	3.07 (3.14)	-2.61 (-11.30–6.08)	0.555
12 months	29	-0.44 (3.09)	28	4.42 (3.17)	-4.86 (-13.58–3.87)	0.274
24 months	26	1.54 (3.19)	24	4.49 (3.30)	-2.95 (-12.00–6.09)	0.520
36 months	21	1.75 (3.39)	21	2.30 (3.43)	-0.56 (-10.05–8.94)	0.908
**NPI hyperactivity (Agitation + Disinhibition + Irritability + Euphoria + Aberrant Motor Behaviour**, **0–60)**						
* Baseline value*	*31*	*3.16 (1.16)*	*29*	*2.29 (1.24)*		
5 months	29	1.17 (1.63)	29	1.83 (1.67)	-0.68 (-5.24–3.93)	0.778
12 months	29	-0.59 (1.63)	28	1.41 (1.68)	-2.00 (-6.61–2.61)	0.393
24 months	26	0.19 (1.68)	24	1.97 (1.77)	-1.79 (-6.59–3.02)	0.465
36 months	21	0.33 (1.79)	21	-0.08 (1.81)	0.40 (-4.61–5.42)	0.874
**NPI psychosis (Delusions + Hallucination + Night-time behaviour**, **0–36)**						
* Baseline value*	*31*	*1.86 (0.73)*	*29*	*2.24 (0.79)*		
5 months	29	0.16 (1.04)	29	-0.14 (1.05)	0.30 (-2.61–3.22)	0.838
12 months	29	0.31 (1.03)	28	0.98 (1.06)	-0.67 (-3.58–2.25)	0.651
24 months	26	-0.20 (1.08)	24	1.74 (1.10)	-1.94 (-4.98–1.10)	0.209
36 months	21	-0.45 (1.13)	21	1.05 (1.15)	-1.50 (-4.67–1.67)	0.353
**NPI affective (Depression + Anxiety**, **0–24)**						
* Baseline value*	*31*	*2.63 (0.63)*	*29*	*1.75 (0.68)*		
5 months	29	-0.22 (0.90)	29	0.31 (0.91)	-0.53 (-3.04–1.98)	0.678
12 months	29	-0.05 (0.90)	28	1.27 (0.92)	-1.32 (-3.84–1.21)	0.305
24 months	26	0.16 (0.92)	24	0.73 (0.96)	-0.57 (-3.18–2.05)	0.668
36 months	21	1.40 (0.98)	21	0.40 (0.99)	1.003 (-1.74–3.75)	0.472
**NPI apathy (Apathy + Appetite**, **0–24)**						
* Baseline value*	*31*	*2.11 (0.73)*	*29*	*2.48 (0.78)*		
5 months	29	-0.65 (1.02)	29	1.07 (1.04)	-1.72 (-4.59–1.15)	0.240
12 months	29	-0.96 (1.02)	28	-0.16 (1.05)	-0.80 (-3.68–2.09)	0.586
24 months	26	1.33 (1.05)	24	0.21 (1.11)	1.12 (-1.89–4.13)	0.465
36 months	21	0.49 (1.12)	21	0.95 (1.13)	-0.45 (-3.59–2.69)	0.777

Linear mixed effect models adjusted for age, sex, and chronic lung disease were used to analyse within- and between-group differences. Higher score represents better function/status, except for Lawton and Brody scale and Neuropsychiatric Inventory

*ADLs *Activities of Daily Living, *SE *Standard error

^a^Change from the baseline value

## Discussion

The present pilot trial, evaluating the feasibility of a person-centred multidimensional and interdisciplinary rehabilitation programme among community-dwelling people with dementia, showed that participants were able to engage in rehabilitation planning, including goal setting. The participants reached a high level of adherence in terms of attendance at exercise and social gatherings in a day rehabilitation unit, as well as high intensity in the exercises. None of the few adverse events during the day rehabilitation unit visits led to a manifest injury or disease. All team professions delivered assessments and interventions during the 20-week rehabilitation period. No significant between-group differences were found in any of the outcomes. However, there seemed to be some potentially clinically meaningful findings in the areas of gait, physical activity, neuropsychiatric symptoms, as well as, being alive and continuing living in the community, warranting consideration of proceeding to a definitive RCT.

All participants, except one, participated in establishing rehabilitation plans. This indicates, despite the aforementioned doubts among healthcare professionals [7], that it is possible for people with dementia to be committed in their rehabilitation. This is essential, as patients’ participation in the rehabilitation process is associated with beneficial treatment effects in older people [58]. Key strategies to enhance participant involvement included facilitating rehabilitation planning in dedicated meetings. During these meetings, participants were actively encouraged by both team staff and informal primary caregivers to articulate their needs and goals. Additionally, a select number of staff, well acquainted with the participant, were chosen to establish a secure and tranquil environment [58].

The participants took part in exercise and other goal-related interventions provided by the team staff to a large extent (Table 2). Contributing factors behind the high adherence, in addition to the valuable support from the informal primary caregiver, might be the strategies used to bridge possible barriers for participation; i.e., organised transports including reception on arrival, reminders, welcoming atmosphere, goal setting, and a person-centred approach [24]. The high adherence may also be explained by the positive experience of the rehabilitation programme, described by interviewed participants in the MIDRED study [31]. Despite an overall high adherence, implementing the rehabilitation programme did not suit all the participants. In a couple of cases, the planned visits at the day rehabilitation unit caused anxiety and sleep disturbance; i.e., Godot syndrome [25]. Unfortunately, that was not noticed at once, since these participants called to cancel with reference to illness.

The exercises, according to the HIFE Program were performed without adverse events leading to any manifest injury or disease, which is consistent with former studies [51, 59, 60]. Participants achieved moderate to high-intensity strength and balance exercises in a high proportion of the attended sessions (86.8% and 98.6%, respectively), which is in accordance with exercise recommendations [61]. In addition, being challenged in exercise has been expressed as mediating motivation to exercise in people with dementia [31, 62, 63]. Similar to previous studies, evaluating the HIFE Program among people with dementia in nursing homes [59, 60], the high-intensity rates were higher for balance exercises than lower-limb exercises.

It can be a challenge to preserve the assessors´ blinding to group allocation when testing people with dementia and their relatives in their homes [64]. However, in the present study, all follow-up assessments were accomplished with preserved blinding, indicating that the pre-established strategy had the intended effect. The few participants who declined assessments were all allocated to the control group and declined mainly at the 24- and 36-month follow-up assessments. Possibly, if the present study had included some attention control activity, the motivation to participate in the follow-up assessments may have increased. Three years is a long follow-up period in the course of dementia, and comorbidities may develop or worsen. Subsequently, several participants may die, as in this sample (*n* = 17). Considering the mortality rate when calculating sample sizes in future randomised controlled trials with long follow-up times in this population is important.

In this pilot trial; i.e., the sample size was not based on a power calculation, there were no statistically significant differences in any of the outcomes. The interventions directed at the control group (usual care), reflecting routine level of care, were relatively few in comparison with the intervention group. Still, it cannot be ruled out that it may have influenced the between-group comparison. However, some findings seemed clinically meaningful. When considering time to event in the survival analyses over 36 months, the intervention group had a lower relative risk for moving to nursing home or mortality compared to the control group. Similar beneficial effects on being alive and living in the community of multi-dimensional assessments and interventions; i.e., comprehensive geriatric assessment (CGA), has previously been reported among older people admitted to hospital [65]. The present trial included people with mild to moderate dementia. However, the opportunities to influence health and prevent moving to nursing homes may have been greater if only mild dementia had been a criterion for inclusion, thus providing interventions earlier in the course of dementia. Furthermore, this outcome was possibly influenced by the interventions addressed to the informal primary caregivers. The information about available societal support might have affected some of the caregivers to apply for nursing homes earlier in the course of dementia. The evaluation of caregivers’ experiences of the intervention, as well as the effects on caregiver burden and quality of life will be addressed in separate papers.

At short-term follow up, between-group differences in gait speed (usual and maximal) approximated statistical significance, and differences favouring the intervention group remained throughout the study period. Even though statistically non-significant, the mean difference between the intervention and the control groups is comparative with reported substantial, meaningful changes, 0.05 m/s and 0.10 m/s in usual and maximal gait speeds, respectively, among older people with moderate mobility disabilities [66]. Moreover, being able to walk short distances backwards is necessary in daily life. A deterioration in backward walking also seems to be associated with an increased risk of falls [67]. Similar to forward gait speed, the non-statistical difference of 0.072 m/s in walking backwards between the intervention and control group at five months seems clinically meaningful. Furthermore, the rehabilitation programme included, in addition to physical exercise, interventions to encourage continuous physical activity. The non-significant between-group differences on physical activity with approximately half an hour per day as well as a reduction of inactivity of 1.5 h per day at 5 months might be clinically meaningful. Over the 36 months, the participants in the intervention group seemed to remain more physically active and spend less time being sedentary compared to the control group. These potential effects might be important in order to maintain physical function during the course of the disease [68] as well as benefiting overall health [69]. Lastly, there were non-significant differences between groups in neuropsychiatric symptoms in favour of the intervention group over the 36-month follow-up (between-group differences of up to 4.9 points in total score of NPI), which may be considered clinically important [70]. It is also noteworthy that the prevalence of neuropsychiatric symptoms in the intervention group had not increased at the five-month assessment, which was performed directly after the rehabilitation period, despite the potential stress it may cause for people with dementia to participate in activities [25].

The clinical meaningfulness of the non-significant between-group difference of 2 points on the Berg balance scale at 5 months is uncertain, but might imply an increased margin to the cut-off score of 45, which indicates a risk of falling and the need of walking aids [39]. The rehabilitation programme did not appear to result in a clinically meaningful effect on nutritional status and ADLs. Interventions targeted explicitly at nutritional status and ADLs were offered individually when needed (Table S.13 and S.8, Supplement, respectively), and could have had a positive impact at the individual level. In addition, other interventions offered could have positively impacted these outcomes, such as physical exercise on ADLs [71] and initiating support at meals by social service at home on nutritional status. However, the interventions explicitly targeting these outcomes were not conducted with regularity and with high intensity among all participants, which might be needed to influence outcomes at the group level positively. Furthermore, cognitive function did not seem to be affected by the rehabilitation programme, despite high attendance at the physical training sessions and potentially beneficial effect on physical activity patterns. Although systematic reviews have shown small to moderate positive effects of physical exercise on cognitive function [72, 73], the results align with large well-conduced RCTs [60, 74, 75]. It might have been beneficial if cognitive training had been part of the rehabilitation programme [76, 77].

A strength of this study is the careful documentation of what was conducted during the intervention period regarding assessments and interventions. The presentation provides an increased understanding of what needs people with dementia in the community may have and facilitates the development and implementation of a larger trial. Despite their progressive condition, participants could complete the majority of the measurements over the follow-up time of 36 months, which suggests they were appropriate for people with dementia. A limitation of the study was that many statistical analyses were performed; i.e., risk of type I error, and thus the results must be interpreted with caution. However, given the multidimensional programme, it was important in this pilot trial to explore potential effects in many common problem areas in dementia separately, which also provides measures of dispersion for future research. The group of participants was heterogeneous regarding dementia type and cognitive function, reflecting the clinical reality in the care of people with dementia but limiting the possibilities of concluding the programme’s feasibility for different types of dementia.

In conclusion, a person-centred multidimensional interdisciplinary rehabilitation programme for community-dwelling people with dementia seems feasible. The potentially clinically meaningful findings on gait, physical activity, and neuropsychiatric symptoms, as well as being alive and continuing to live in the community after 36 months, suggest that the rehabilitation programme is worthwhile to evaluate in an adequately powered RCT, including cost-effectiveness calculation. One could consider conducting the programme earlier in the course of dementia, adding cognitive training and an attention control activity.

## Supplementary Information

Additional file 1. Table A. 1-20. Type of assessments and interventions per profession, addressed to participants with dementia in the intervention group.

## Data Availability

The datasets generated and/or analysed during the current study are not publicly available due to issues of individual privacy but are available from the corresponding author on reasonable request.
