# Supplementary material for: Feasibility of a person-centred multidimensional interdisciplinary rehabilitation programme in community-dwelling people with dementia: a randomised controlled pilot trial
Source: BMC Geriatr. 2024 Sep 28;24:794. doi: 10.1186/s12877-024-05372-9 (PMC11439292; doi:10.1186/s12877-024-05372-9)
Supplement: Supplementary file 1 — Additional file 1. Table A. 1-20. Type of assessments and interventions per profession, addressed to participants with dementia in the intervention group. [file 12877_2024_5372_MOESM1_ESM.docx]

**Additional file 1.** Feasibility of a person-centred multidimensional interdisciplinary rehabilitation programme in community-dwelling people with dementia: a randomised controlled pilot trial.

**Table A. 1-20. Type of assessments and interventions**^a^ **per profession, addressed to participants with dementia in the intervention group.**

A.1. Assistant nurse, assessments

| **Assessments** | **Rehabilitation period, twenty weeks n=31** | | **5-month follow-up, four weeks, n=29** | | **14-month follow-up, four weeks, n=27** | |
| --- | --- | --- | --- | --- | --- | --- |
|  | Participants receiving assessment, n (%) | Assessments per participant assessed  Median (IQR^b^) | Participants receiving assessment, n (%) | Assessments per participant assessed  Median (IQR) | Participants receiving assessment, n (%) | Assessments per participant assessed  Median (IQR) |
| Blood pressure | 3 (9.7) | 2 (1.0–2.0) | 0 | 0 | 0 | 0 |
| Electrocardiography | 2 (6.5) | 1 (1.0–1.0) | 0 | 0 | 0 | 0 |
| Blood sample | 3 (9.7) | 1 (1.0–1.0) | 0 | 0 | 0 | 0 |
| **Total** | 6 (19.4) | 1.5 (1.0–2.25) | 0 | 0 | 0 | 0 |

A.2. Assistant nurse, interventions

| **Interventions** | **Rehabilitation period, twenty weeks n=31** | | **5-month follow-up, four weeks, n=29** | | **14-month follow-up, four weeks, n=27** | |
| --- | --- | --- | --- | --- | --- | --- |
|  | Participants receiving intervention, n (%) | Interventions per participant receiving intervention  Median (IQR) | Participants receiving intervention, n (%) | Interventions per participant receiving intervention  Median (IQR) | Participants receiving intervention, n (%) | Interventions per participant receiving intervention Median (IQR) |
| Dressing wounds | 1 (3.2) | 2 (2.0–2.0) | 0 | 0 | 0 | 0 |
| Support participation in exercise groups in community | 0 | 0 | 0 | 0 | 1 (3.7) | 1 (1.0–1.0) |
| **Total** | 1 (3.2) | 2 (2.0–2.0) | 0 | 0 | 1 (3.7) | 1 (1.0–1.0) |

A.3. Clinical pharmacist, assessments

| **Assessments** | **Rehabilitation period, twenty weeks n=31** | | **5-month follow-up, four weeks, n=29** | | **14-month follow-up, four weeks, n=27** | |
| --- | --- | --- | --- | --- | --- | --- |
|  | Participants receiving assessment, n (%) | Assessments per participant assessed  Median (IQR) | Participants receiving assessment, n (%) | Assessments per participant assessed  Median (IQR) | Participants receiving assessment, n (%) | Assessments per participant assessed  Median (IQR) |
| Comprehensive medication review | 30 (96.8) | 1 (1.0–1.0) | 28 (96.6) | 1 (1.0–1.0) | 27 (100) | 1 (1.0–1.0) |
| **Total** | 30 (96.8) | 1 (1.0–1.0) | 28 (96.6) | 1 (1.0–1.0) | 27 (100) | 1 (1.0–1.0) |

A.4. Clinical pharmacist, interventions

| **Intervention** | **Rehabilitation period, twenty weeks n=31** | | **5-month follow-up, four weeks, n=29** | | **14-month follow-up, four weeks, n=27** | |
| --- | --- | --- | --- | --- | --- | --- |
|  | Participants receiving intervention, n (%) | Interventions per participant receiving intervention  Median (IQR) | Participants receiving intervention, n (%) | Interventions per participant receiving intervention  Median (IQR) | Participants receiving intervention, n (%) | Interventions per participant receiving intervention  Median (IQR) |
| Advice and information about medication | 9 (29.0) | 1 (1.0–1.0) | 1 (3.4) | 1 (1.0–1.0) | 1 (3.7) | 1 (1.0–1.0) |
| **Total** | 9 (29.0) | 1 (1.0–1.0) | 1 (3.4) | 1 (1.0–1.0) | 1 (3.7) | 1 (1.0–1.0) |

A.5. Dental hygienist, assessments

| **Assessments** | **Rehabilitation period, twenty weeks, n=31** | | **5-month follow-up, four weeks, n=29** | | **14-month follow-up, four weeks, n=27** | |
| --- | --- | --- | --- | --- | --- | --- |
|  | Participants receiving assessment, n (%) | Assessments per participant assessed  Median (IQR) | Participants receiving assessment, n (%) | Assessments per participant assessed  Median (IQR) | Participants receiving assessment, n (%) | Assessments per participant assessed  Median (IQR) |
| Clinical examination of mouth and teeth | 30 (96.8) | 1 (1.0–1.0) | 29 (100) | 1 (1.0–1.0) | 27 (100) | 1 (1.0–1.0) |
| **Total** | 30 (96.8) | 1 (1.0–1.0) | 29 (100) | 1 (1.0–1.0) | 27 (100) | 1 (1.0–1.0) |

A.6. Dental hygienist, interventions

| **Interventions** | **Rehabilitation period, twenty weeks n=31** | | **5-month follow-up, four weeks, n=29** | | **14-month follow-up, four weeks, n=27** | |
| --- | --- | --- | --- | --- | --- | --- |
|  | Participants receiving intervention, n (%) | Interventions per participant receiving intervention  Median (IQR) | Participants receiving intervention, n (%) | Interventions per participant receiving intervention  Median (IQR) | Participants receiving intervention, n (%) | Interventions per participant receiving intervention  Median (IQR) |
| Training in oral care | 17 (54.8) | 1 (1.0–1.0) | 0 | 0 | 0 | 0 |
| Advice concerning oral care | 30 (96.8) | 2 (1.0–2.0) | 28 (96.6) | 1 (1.0–1.0) | 27 (100) | 1 (1.0–1.0) |
| **Total** | 30 (96.8) | 3 (2.0–4.0) | 28 (96.6) | 1 (1.0–1.0) | 27 (100) | 1 (1.0–1.0) |

A.7. Dietician, assessments

| **Assessments** | **Rehabilitation period, twenty weeks n=31** | | **5-month follow-up, four weeks, n=29** | | **14-month follow-up, four weeks, n=27** | |
| --- | --- | --- | --- | --- | --- | --- |
|  | Participants receiving assessment, n (%) | Assessments per participant assessed  Median (IQR) | Participants receiving assessment, n (%) | Assessments per participant assessed  Median (IQR) | Participants receiving assessment, n (%) | Assessments per participant assessed  Median (IQR) |
| Nutritional intake | 30 (96.8) | 1 (1.0–1.0) | 29 (100) | 1 (1.0–1.0) | 27 (100) | 1 (1.0–1.0) |
| Body height | 28 (90.3) | 1 (1.0–1.0) | 1 (3.4) | 1 (1.0–1.0) | 0 | 0 |
| Body weight | 30 (96.8) | 5 (4.75–6.0) | 27 (93.1) | 1 (1.0–1.0) | 24 (88.9) | 1 (1.0–1.0) |
| **Total** | 30 (96.8) | 7 (6.75–8.0) | 29 (100) | 2 (2.0–2.0) | 27 (100) | 2 (2.0–2.0) |

A.8. Dietician, interventions

| **Interventions** | **Rehabilitation period, twenty weeks n=31** | | **5-month follow-up, four weeks, n=29** | | **14-month follow-up, four weeks, n=27** | |
| --- | --- | --- | --- | --- | --- | --- |
|  | Participants receiving intervention, n (%) | Interventions per participant receiving intervention  Median (IQR) | Participants receiving intervention, n (%) | Interventions per participant receiving intervention  Median (IQR) | Participants receiving intervention, n (%) | Interventions per participant receiving intervention  Median (IQR) |
| Dietary enrichment | 7 (22.6) | 2 (1.0–3.25) | 0 | 0 | 1 (3.7) | 1 (1.0–1.0) |
| Advice concerning eating habits | 5 (16.1) | 1 (1.0–1.5) | 2 (6.9) | 1 (1.0–1.0) | 0 | 0 |
| **Total** | 8 (25.8) | 2.5 (1.0–3.75) | 2 (6.9) | 1 (1.0–1.0) | 1 (3.7) | 1 (1.0–1.0) |

A.9. Neuropsychologist, assessments

| **Assessments** | **Rehabilitation period, twenty weeks n=31** | | **5-month follow-up, four weeks, n=29** | | **14-month follow-up, four weeks, n=27** | |
| --- | --- | --- | --- | --- | --- | --- |
|  | Participants receiving assessment, n (%) | Assessments per participant assessed  Median (IQR) | Participants receiving assessment, n (%) | Assessments per participant assessed  Median (IQR) | Participants receiving assessment, n (%) | Assessments per participant assessed  Median (IQR) |
| Emotional functions* | 7 (22.6) | 1 (1.0–1.0) | 3 (10.3) | 1 (1.0–1.0) | 2 (7.4) | 1 (1.0–1.0) |
| **Total** | 7 (22.6) | 1 (1.0–1.0) | 3 (10.3) | 1 (1.0–1.0) | 2 (7.4) | 1 (1.0–1.0) |

* Investigation of the participants' ability to understand and manage their emotions

A.10. Neuropsychologist, interventions

| **Intervention** | **Rehabilitation period, twenty weeks n=31** | | **5-month follow-up, four weeks, n=29** | | **14-month follow-up, four weeks, n=27** | |
| --- | --- | --- | --- | --- | --- | --- |
|  | Participants receiving intervention, n (%) | Interventions per participant receiving intervention  Median (IQR) | Participants receiving intervention, n (%) | Interventions per participant receiving intervention  Median (IQR) | Participants receiving intervention, n (%) | Interventions per participant receiving intervention  Median (IQR) |
| Supporting | 7 (22.6) | 2 (1.0–4.0) | 3 (10.3) | 1 (1.0–1.0) | 2 (7.4) | 1 (1.0–1.0) |
| Advice and information | 2 (6.5) | 1 (1.0–1.0) | 0 | 0 | 0 | 0 |
| **Total** | 8 (25.8) | 2 (1.0–4.0) | 3 (10.3) | 1 (1.0–1.0) | 2 (7.4) | 1 (1.0–1.0) |

A.11. Nurse, assessments

| **Assessments** | **Rehabilitation period, twenty weeks n=31** | | **5-month follow-up, four weeks, n=29** | | **14-month follow-up, four weeks, n=27** | |
| --- | --- | --- | --- | --- | --- | --- |
|  | Participants receiving assessment, n (%) | Assessments per participant assessed  Median (IQR) | Participants receiving assessment, n (%) | Assessments per participant assessed  Median (IQR) | Participants receiving assessment, n (%) | Assessments per participant assessed  Median (IQR) |
| Blood pressure | 1 (3.2) | 2 (2.0–2.0) | 0 | 0 | 0 | 0 |
| Blood sample | 1 (3.2) | 1 (1.0–1.0) | 0 | 0 | 0 | 0 |
| Investigation of mental functions | 1 (3.2) | 1 (1.0–1.0) | 1 (3.4) | 1 (1.0–1.0) | 0 | 0 |
| Clinical examination of conditions which arose during the rehabilitation program | 4 (12.9) | 1 (1.0–2.5) | 1 (3.4) | 1 (1.0–1.0) | 0 | 0 |
| **Total** | 4 (12.9) | 1 (1.0–4.75) | 2 (6.9) | 1 (1.0–1.0) | 0 | 0 |

A.12. Nurse, interventions

| **Intervention** | **Rehabilitation period, twenty weeks n=31** | | **5-month follow-up, four weeks, n=29** | | **14-month follow-up, four weeks, n=27** | |
| --- | --- | --- | --- | --- | --- | --- |
|  | Participants receiving intervention, n (%) | Interventions per participant receiving intervention  Median (IQR) | Participants receiving intervention, n (%) | Interventions per participant receiving intervention  Median (IQR) | Participants receiving intervention, n (%) | Interventions per participant receiving intervention  Median (IQR) |
| Attestation | 15 (48.4) | 1 (1.0–1.0) | 0 | 0 | 0 | 0 |
| Advice and information about medication | 2 (6.5) | 2 (1.0–2.0) | 0 | 0 | 0 | 0 |
| Dressing wounds | 0 | 0 | 1 (3.4) | 2 (2.0–2.0) | 0 | 0 |
| Supporting informal caregiver | 1 (3.2) | 1 (1.0–1.0) | 1 (3.4) | 1 (1.0–10.) | 0 | 0 |
| **Total** | 16 (51.6) | 1 (1.0–1.0) | 2 (6.9) | 1.5 (1.0–1.5) | 0 | 0 |

A.13. Occupational therapist, assessments

| **Assessments** | **Rehabilitation period, twenty weeks n=31** | | **5-month follow-up, four weeks, n=29** | | **14-month follow-up, four weeks, n=27** | |
| --- | --- | --- | --- | --- | --- | --- |
|  | Participants receiving assessment, n (%) | Assessments per participant assessed  Median (IQR) | Participants receiving assessment, n (%) | Assessments per participant assessed  Median (IQR) | Participants receiving assessment, n (%) | Assessments per participant assessed  Median (IQR) |
| Cognitive functions | 2 (6.5) | 1 (1.0–1.0) | 1 (3.4) | 1 (1.0–1.0) | 0 | 0 |
| General tasks and demands | 21 (67.7) | 1 (1.0–1.0) | 1 (3.4) | 1 (1.0–1.0) | 0 | 0 |
| Communication | 2 (6.5) | 1 (1.0–1.0) | 0 | 0 | 0 | 0 |
| Mobility | 5 (16.1) | 1 (1.0–1.0) | 5 (17.2) | 1 (1.0–1.0) | 11 (40.7) | 1 (1.0–1.0) |
| Self-care | 30 (96.8) | 1 (1.0–1.0) | 29 (100) | 1 (1.0–1.0) | 27 (100) | 1 (1.0–1.0) |
| Domestic life | 30 (96.8) | 1 (1.0–1.0) | 27 (93.1) | 1 (1.0–1.0) | 27 (100) | 1 (1.0–1.0) |
| Community, social and civic life | 29 (93.5) | 1 (1.0–1.0) | 27 (93.1) | 1 (1.0–1.0) | 26 (96.3) | 1 (1.0–1.0) |
| Environmental factors | 31 (100) | 2 (1.0–2.0) | 17 (58.6) | 1 (1.0–1.5) | 15 (55.6) | 1 (1.0–2.0) |
| **Total** | 31 (100) | 6 (6.0–7.0) | 29 (100) | 4 (3.0–5.0) | 27 (100) | 4 (4.0–5.0) |

A.14. Occupational therapist, interventions

| **Interventions** | **Rehabilitation period, twenty weeks, n=31** | | **5-month follow-up, four weeks, n=29** | | **14-month follow-up, four weeks, n=27** | |
| --- | --- | --- | --- | --- | --- | --- |
|  | Participants receiving intervention, n (%) | Interventions per participant receiving intervention  Median (IQR) | Participants receiving intervention, n (%) | Interventions per participant receiving intervention  Median (IQR) | Participants receiving intervention, n (%) | Interventions per participant receiving intervention  Median (IQR) |
| Cognitive functions | 1 (3.2) | 1 (1.0–1.0) | 1 (3.4) | 1 (1.0–1.0) | 0 | 0 |
| Falls prevention | 2 (6.5) | 1 (1.0–1.0) | 1 (3.4) | 1 (1.0–1.0) | 0 | 0 |
| Communication | 1 (3.2) | 1 (1.0–1.0) | 0 | 0 | 0 | 0 |
| Mobility | 1 (3.2) | 1 (1.0–1.0) | 1 (3.4) | 1 (1.0–1.0) | 1 (3.7) | 1 (1.0–1.0) |
| Self-care | 3 (9.7) | 1 (1.0–1.0) | 0 | 0 | 0 | 0 |
| Domestic life | 5 (16.1) | 1 (1.0–4.5) | 1 (3.4) | 2 (2.0–2.0) | 0 | 0 |
| Supporting informal primary caregivers´ participation in daily care | 2 (6.5) | 1 (1.0–1.0) | 0 | 0 | 0 | 0 |
| Support participation in exercise groups in community | 1 (3.2) | 1 (1.0–1.0) | 1 (3.4) | 1 (1.0–1.0) | 0 | 0 |
| Community, social and civic life | 8 (25.8) | 2 (1.0–2.0) | 0 | 0 | 0 | 0 |
| Environmental factors;  Adaptation of environment including assistive devices  Cognitive devices | 26 (83.9)  20 (64.5) | 3 (2.0–5.0)  2 (1.0–4.0) | 8 (27.6)  12 (41.4) | 1 (1.0–1.0)  1 (1.0–1.75) | 9 (33.3)  9 (33.3) | 2 (1.5–3.0)  1 (1.0–1.5) |
| **Total** | 28 (90.3) | 3 (2.0–5.0) | 18 (62.1) | 1 (1.0–3.0) | 15 (55.6) | 2 (1.0–3.0) |

A.15. Physical therapist, assessments

| **Assessments** | **Rehabilitation period, twenty weeks n=31** | | **5-month follow-up, four weeks, n=29** | | **14-month follow-up, four weeks, n=27** | |
| --- | --- | --- | --- | --- | --- | --- |
|  | Participants receiving assessment, n (%) | Assessments per participant assessed  Median (IQR) | Participants receiving assessment, n (%) | Assessments per participant assessed  Median (IQR) | Participants receiving assessment, n (%) | Assessments per participant assessed  Median (IQR) |
| Cognitive functions | 5 (16.1) | 1 (1.0–1.5) | 0 | 0 | 1 (3.7) | 1 (1.0–1.0) |
| Balance and risk of falling | 30 (96.8) | 2 (1.0–3.0) | 29 (100) | 1 (1.0–1.0) | 26 (96.3) | 1 (1.0–1.0) |
| Cardio-respiratory function | 13 (31.9) | 2 (2.0–5.0) | 1 (3.4) | 1 (1.0–1.0) | 1 (3.7) | 1 (1.0–1.0) |
| Joint and muscle function | 20 (64.5) | 2 (1.0–2.75) | 28 (96.6) | 1 (1.0–1.0) | 26 (96.3) | 1 (1.0–1.0) |
| Mobility | 30 (96.8) | 2 (1.0–2.0) | 29 (100) | 2 (1.0–2.0) | 27 (100) | 1 (1.0–2.0) |
| Environmental factors | 4 (12.9) | 1 (1.0–1.0) | 1 (3.4) | 1 (1.0–1.0) | 1 (3.7) | 1 (1.0–1.0) |
| **Total** | 30 (96.8) | 7 (4.0–9.0) | 29 (100) | 4 (3.0–4.0) | 27 (100) | 3 (3.0–4.0) |

A.16. Physical therapist, interventions

| **Interventions** | **Rehabilitation period, twenty weeks, n=31** | | **5-month follow-up, four weeks, n=29** | | **14-month follow-up, four weeks, n=27** | |
| --- | --- | --- | --- | --- | --- | --- |
|  | Participants receiving intervention, n (%) | Interventions per participant receiving intervention  Median (IQR) | Participants receiving intervention, n (%) | Interventions per participant receiving intervention  Median (IQR) | Participants receiving intervention, n (%) | Interventions per participant receiving intervention  Median (IQR) |
| Exercise related to balance and falls prevention | 30 (96.8) | 26 (20.5–28.25) | 2 (6.9) | 1 (1.0–1.0) | 3 (11.1) | 1 (1.0–1.0) |
| Exercise related to cardio-respiratory function | 4 (12.9) | 2 (1.25–8.75) | 0 | 0 | 0 | 0 |
| Exercise related to joint function and leg muscle strength | 30 (96.8) | 27 (20.5–29.0) | 0 | 0 | 1 (3.7) | 1 (1.0–1.0) |
| Exercise related to transferring and gait (Mobility) | 6 (19.4) | 1 (1.0–1.25) | 0 | 0 | 1 (3.7) | 1 (1.0–1.0) |
| Prescription of assistive devices | 6 (19.4) | 1.5 (1.0–2.5) | 3 (10.3) | 1 (1.0–1.0) | 2 (7.4) | 1 (1.0–1.0) |
| Prescription of exercise program | 11 (35.5) | 2 (1.0–2.0) | 8 (27.6) | 1.5 (1.0–2.0) | 10 (37.0) | 2 (2.0–2.0) |
| Motivational interviewing about physical activity | 9 (29.0) | 1 (1.0–2.0) | 3 (10.3) | 1 (1.0–1.0) | 6 (22.2) | 1 (1.0–1.25) |
| Support participation in exercise groups or gym in the community | 3 (9.7) | 1 (1.0–1.0) | 6 (20.7) | 1 (1.0–2.0) | 4 (14.8) | 1 (1.0–1.0) |
| **Total** | 30 (96.8) | 55 (42.0–60.0) | 18 (62.1) | 1 (1.0–2.0) | 17 | 2 (1.0–3.0) |

A.17. Physician, assessments

| **Assessments** | **Rehabilitation period, twenty weeks n=31** | | **5-month follow-up, four weeks, n=29** | | **14-month follow-up, four weeks, n=27** | |
| --- | --- | --- | --- | --- | --- | --- |
|  | Participants receiving assessment, n (%) | Assessments per participant assessed  Median (IQR) | Participants receiving assessment, n (%) | Assessments per participant assessed  Median (IQR) | Participants receiving assessment, n (%) | Assessments per participant assessed  Median (IQR) |
| Clinical examination of general health | 30 (96.8) | 1 (1.0–1.0) | 26 (89.7) | 1 (1.0–1.0) | 27 (100) | 1 (1.0–1.0) |
| Clinical examination of cardiovascular and respiratory function | 25 (80.6) | 1 (1.0–1.0) | 2 (6.9) | 1 (1.0–1.0) | 3 (11.1) | 1 (1.0–1.0) |
| Cognitive functions | 29 (93.5) | 1 (1.0–1.0) | 17 (58.6) | 1 (0–1.0) | 8 (29.6) | 1 (1.0–1.0) |
| Clinical examination of medical conditions which arose during the rehabilitation program | 6 (19.4) | 1 (1.0–1.25) | 3 (10.3) | 1 (1.0–1.0) | 0 | 0 |
| **Total** | 30 (96.8) | 3 (3.0–4.0) | 28 (96.6) | 2 (1.0–2.0) | 27 (100) | 1 (1.0–2.0) |

A.18. Physician, interventions

| **Intervention** | **Rehabilitation period, twenty weeks n=31** | | **5-month follow-up, four weeks, n=29** | | **14-month follow-up, four weeks, n=27** | |
| --- | --- | --- | --- | --- | --- | --- |
|  | Participants receiving intervention, n (%) | Interventions per participant receiving intervention  Median (IQR) | Participants receiving intervention, n (%) | Interventions per participant receiving intervention  Median (IQR) | Participants receiving intervention, n (%) | Interventions per participant receiving intervention  Median (IQR) |
| Prescription of medication | 9 (29.0) | 1 (1.0–1.5) | 5 (17.2) | 1 (1.0–1.0) | 2 (7.4) | 1 (1.0–1.0) |
| Prescription renewal | 6 (19.4) | 1 (1.0–1.0) | 1 (3.4) | 1 (1.0–1.0) | 1 (3.7) | 1 (1.0–1.0) |
| Decision making on referral or test results | 2 (6.5) | 3 (2.0–3.0) | 1 (3.4) | 1 (1.0–1.0) | 0 | 0 |
| Advice and information about medication | 3 (9.7) | 1 (1.0–1.0) | 0 | 0 | 0 | 0 |
| Remittance | 6 (19.4) | 1 (1.0–1.0) | 2 (6.9) | 1 (1.0–1.0) | 4 (14.8) | 1 (1.0–1.0) |
| Attestation | 1 (3.2) | 1 (1.0–1.0) | 0 |  | 1 (3.7) | 1 (1.0–1.0) |
| **Total** | 16 (51.6) | 1 (1.0–2.0) | 9 (31.0) | 1 (1.0–1.0) | 8 (29.6) | 1 (1.0–1.0) |

A.19. Social worker, assessments

| **Assessments** | **Rehabilitation period, twenty weeks n=31** | | **5-month follow-up, four weeks, n=29** | | **14-month follow-up, four weeks, n=27** | |
| --- | --- | --- | --- | --- | --- | --- |
|  | Participants receiving assessment, n (%) | Assessments per participant assessed  Median (IQR) | Participants receiving assessment, n (%) | Assessments per participant assessed  Median (IQR) | Participants receiving assessment, n (%) | Assessments per participant assessed  Median (IQR) |
| Informal care-givers´ burden | 30 (96.8) | 1 (1.0-1.0) | 29 (100) | 1 (1.0-1.0) | 27 (100) | 1 (1.0-1.0) |
| Community, social and civic life | 1 (3.2) | 1 (1.0–1.0) | 0 | 0 | 0 | 0 |
| **Total** | 30 (96.8) | 1 (1.0–1.25) | 29 (100) | 1 (1.0–1.0) | 27 (100) | 1 (1.0–1.0) |

A.20. Social worker, interventions

| **Interventions** | **Rehabilitation period, twenty weeks, n=31** | | **5-month follow-up, four weeks, n=29** | | **14-month follow-up, four weeks, n=27** | |
| --- | --- | --- | --- | --- | --- | --- |
|  | Participants receiving intervention, n (%) | Interventions per participant receiving intervention  Median (IQR) | Participants receiving intervention, n (%) | Interventions per participant receiving intervention  Median (IQR) | Participants receiving intervention, n (%) | Interventions per participant receiving intervention  Median (IQR) |
| Psychological support | 12 (38.7) | 1 (1.0–2.0) | 2 (6.9) | 1 (1.0–1.0) | 3 (11.1) | 1 (1.0–1.0) |
| Support in economic life | 3 (9.7) | 2 (1.0–2.0) | 1 (3.4) | 2 (2.0–2.0) | 1 (3.7) | 3 (3.0–3.0) |
| Support and training in community, social and civic life | 1 (3.2) | 1 (1.0–1.0) | 0 | 0 | 0 | 0 |
| Support participation in exercise groups in community | 3 (9.7) | 1 (1.0–1.0) | 0 | 0 | 0 | 0 |
| Attestation | 4 (12.9) | 1 (1.0–1.0) | 2 (6.9) | 1.5 (1.0–1.5) | 4 (14.8) | 1 (1.0–1.0) |
| **Total** | 31 (100) | 7 (5.0–9.0) | 28 (96.5) | 1.0 (1.0–3.0) | 27 (100) | 1 (1.0–2.0) |

^a^Each assessment and intervention, documented in medical records, was coded based on the National Board of Health´s Classification of care measures and interventtions [1]. Each contact with participant could include more than one assessment/intervention.

^b^IQR=interquartile range

Reference:

1. The National Board of Health and Welfare. [Klassifikation av vårdåtgärder.] Classification of care measures and interventions. (Publications in Swedish). <https://www.socialstyrelsen.se/statistik-och-data/klassifikationer-och-koder/kva/>
